# Intraoperative parathyroid hormone level in parathyroidectomy: which patients benefit from it?

**DOI:** 10.1186/1916-0216-42-56

**Published:** 2013-12-19

**Authors:** Faisal Zawawi, Alex M Mlynarek, Arielle Cantor, Rickul Varshney, Martin J Black, Michael P Hier, Louise Rochon, Richard J Payne

**Affiliations:** 1 Department of Otolaryngology, Head and Neck Surgery, McGill University, Room E3-37, 687 Pine Avenue West, Montreal, QC H3A 1A1, Canada; 2 Faculty of Medicine, McGill University, Montreal, QC, Canada; 3 Department of Pathology, McGill University, Montreal, QC, Canada; 4 King Abdulaziz University, Jeddah, Saudi Arabia

**Keywords:** Parathyroidectomy, Intraoperative, Frozen section, PTH

## Abstract

**Background:**

Intraoperative parathyroid hormone level (IOPTH) is withdrawn during parathyroidectomy to confirm the success of the procedure. Recently, the importance of IOPTH has been put to question. The purpose of this study is to determine whether IOPTH is necessary for all patients undergoing parathyroidectomy in the presence of frozen section.

**Materials and methods:**

A cohort study of parathyroidectomies was performed in three university affiliated hospitals during 2007-2012. The patients were divided into two groups. Group 1: Patients with two preoperative concordant imaging localizing a hyperactive gland. Group 2: Patients without two concordant imaging. A comparison of benefit of IOPTH was carried out. Frozen section results were also analyzed to determine sensitivity and predictability of a parathyroid adenoma.

**Results:**

The study considered 221 patients having parathyroidectomies for primary hyperparathyroidism (PHPT). Of them, 10 were excluded due to incomplete data. Among the remaining, 186 had 2 concordant imaging preoperatively localizing an adenoma. 93.5% of whom were found intraoperatively in that location. IOPTH was not found to be of importance in 98.92% of the preoperative localized adenomas in the presence of frozen section. IOPTH added an estimate of 30.9 minutes on average to the surgery time.

**Conclusion:**

This study demonstrates that the added operating time associated with IOPTH may not be justified for patients undergoing parathyroidectomy who have 2 concordant imaging preoperatively in the presence of frozen section. This study suggests a simple algorithm, The McGill Parathyroid Protocol (MPP), to help in approaching PHPT patients undergoing parathyroidectomy.

## Introduction

The incidence of primary hyperparathyroidism (PHPT) is less than 1% [1]. The most common cause of PHPT is a sporadic solitary adenoma (90%) [2-4]. Four-gland hyperplasia (9%) and double adenomas (3%) are less prevalent causes [2-4]. Surgical excision of the pathological gland(s), for the patients requiring treatment for PHPT, is the current standard of the care [4]. Traditionally, surgical excision included parathyroidectomy with bilateral neck exploration. However, in recent years, this approach has been replaced in many centers by a more focused technique involving minimal dissection. This transition is partly attributed to the evolution of perioperative diagnostic modalities [5,6].

Currently, most PHPT patients are assessed by Technetium (Tc) 99 m sestamibi (MIBI) scan, ultrasound (US), and possibly magnetic resonance imaging (MRI) [5]. Some surgeons use frozen section (FS) to identify the tissue that is being excised prior to concluding the procedure. Pathologists can identify the hyperactive gland on frozen section by comparing it with a normal looking parathyroid or other tissues [7].

Intraoperative parathyroid hormone level (IOPTH), adjunct to imaging and frozen section, has been used in many tertiary centers to confirm the removal of the hyperactive gland [8]. Although IOPTH helps in confirming the adenoma excision, but it can be time consuming and also requires significant resources [9]. Recently, few reports questioned the benefits of using IOPTH for all patients undergoing parathyroidectomy, and suggested that the importance of IOPTH diminishes if the parathyroid adenoma is localized by two or more concordant imaging modalities [4,9,10].

This study is aimed to improve our understanding regarding the importance of IOPTH during parathyroidectomy and to determine whether its implication on routine bases in all cases is justified, considering the availability of limited resources in current healthcare system.

## Materials and methods

This study was approved by the Institutional review board of McGill University.

### Population

This is a 6-year cohort study conducted in two phases at The McGill University Thyroid Cancer Center during January 2007 to December 2012. The first phase consisted of a 5-year retrospective chart review. The second phase was a 1-year prospective observation study. The data from both phases were collected and analyzed together. The studied population consisted patients undergoing parathyroidectomies for primary hyperparathyroidism. Exclusion criteria included patients with missing data due to lack of documentation and patients lacking the proper workup (no IOPTH, no frozen section, or no preoperative 24 h urine calcium).

The second, prospective phase, was added to strengthen the study by increasing the sample size. Both of the phases used the same criteria for the data collection. Additionally, to reduce potential bias in prospective data collection the pathologists, radiologists, nurses, and laboratory technicians were all blinded.

### Preoperative assessment

The data collection for the patients, who were referred to the McGill University Thyroid Cancer Center for a suspected or confirmed PHPT, began by having a detailed history and physical examination performed with a focus on hypercalcemia signs and symptoms. Laboratory testing included PTH assays, serum electrolytes, and 24-hour urine calcium to rule out Familial Hypocalciuric Hypercalcemia.

During the investigation period, the patients were imaged by two modalities, usually ultrasound and MIBI scan. If the imaging modalities were not concordant, a third modality, most often MRI, was requested. After appropriate localization, a decision between the surgeon, the endocrinologist, and the patient was made regarding the need for surgery.

### The procedure

After the patient was intubated, serum PTH level was measured prior to initiation of the surgery. Under general anesthesia, the surgeon performed a minimal dissection at the localized area; and the suspected adenoma was sent for frozen section (FS) analysis. Meanwhile, the surgeon explored the other ipsilateral parathyroid gland. At 12 minutes of mark post-excision, IOPTH was analyzed using a Cobas^©^ 8000 machine. The confirmation of adequate excision of the adenoma was based on the FS and IOPTH results. If the FS showed hypercellular gland or adenoma and the IOPTH dropped by 50% from the pre-excision level, the surgery was deemed adequate. If the IOPTH did not drop, the surgeon explored the neck seeking another adenoma and if found necessary, a 4-gland exploration was performed. Post-operatively, a 1-hour PTH value was also measured.

### Data collection and analysis

The following data were collected for every patient: demographics, preoperative PTH, preoperative imaging (ultrasound, MIBI, MRI, CT Scan, and PET scan), intraoperative findings, IOPTH, FS results, wait time for IOPTH, wait time for FS, final pathology, accuracy of preoperative imaging in concordance to intraoperative findings and postoperative PTH levels.

The results were then plotted electronically and analyzed using SPSS^©^ 20.0. Descriptive statistics were performed alongside measuring the sensitivity and specificity of FS. Positive Predictive Values (PPV) and Negative Predictive Values (NPV) were calculated. Pearson Chi-Square was used to detect statistical difference in benefit of IOPTH among patients depending on their preoperative imaging localization status. While Fisher exact test was used to determine significance among frozen section results and its ability to detect a hyperactive gland.

### Definitions

Localized preoperatively: A suspected adenoma that has two concordant preoperative imaging studies localizing the adenoma at a specific location.

Accurate Localization: The preoperative localization is representative to the location of the adenoma intraoperatively.

Useful IOPTH: IOPTH result leading to a change in management.

Wait time for IOPTH: Time interval between adenoma excision and the result of IOPTH.

Wait time for FS: Time interval between adenoma excisions and the FS result.

## Results

### Demographics

A total of 221 patients had parathyroidectomies for PHPT at the McGill University Thyroid Cancer Center during January 2007 to December 2012. Ten patients were excluded from this study due to incomplete documentation. Out of 211 patients, 161 were females (76.3%) and the mean age of the population was 57.7 years.

### Imaging

The patients were divided into two groups; patients who localized preoperatively by two concordant imaging (186/211) and patients who did not have two concordant imaging (25/211).

186/211 patients localized preoperatively (88.15%) by two concordant imaging (nuclear or radiological) modalities. In this group, 174/186 patients (93.5%) had accurate localization. 25/211 patients did not have two concordant imaging modalities preoperatively (11.85%). In these patients, MIBI was more predictive for the location of the hyperactive gland compared to Ultrasound. The PPV of MIBI in this group of patients was 92.85%.

Considering both the groups, 12/211 patients had imaging suggestive of multiple pathological glands. Of those 12, on final pathology, 7 patients had parathyroid gland hyperplasia, 3 patients had double adenomas, and 2 patients had solitary adenoma. In all these instances, ultrasound and MRI had PPV of 90% and 100%, respectively.

### Frozen section and final pathology

For the 211 patients included in this study, 232 frozen sections were performed (Table 1). The sensitivity of FS for detecting an adenoma was 96.74% and the specificity was 81.25%. The positive predictive value (PPV) was 95.19%, and the negative predictive value (NPV) was 86.67%. FS was able to diagnose hyperplasia in 10 patients (52.6%). Hypercellular parathyroid tissue in FS was associated with an adenoma outcome in 111 patients (92.5% of hypercellular FS).

**Table 1:** This table lists the 5 different frozen section encountered in this study and their final pathology results

**FS result**	**Final pathology result**				
	**Adenoma**	**Hyperplasia**	**Normal parathyroid**	**No parathyroid**	**Total**
Hypercellular	111	9	0	0	120
Adenoma	67	0	0	0	67
Hyperplasia	3	10	0	0	13
Normal parathyroid tissue	3	0	21	0	24
No parathyroid tissue	0	0	0	8	8
Total	184	19	21	8	232

There was no difference in adenoma outcome in final pathology between the group that had Hypercellular FS and Adenoma FS (p value = 0.168). The PPV of Frozen Section to Adenoma is 95.19% and NPV is 86.67%.

Double adenomas were present in 6 patients (2.8%) and hyperplasia was diagnosed in 19 patients (9%) (Table 1).

**Figure 1:**
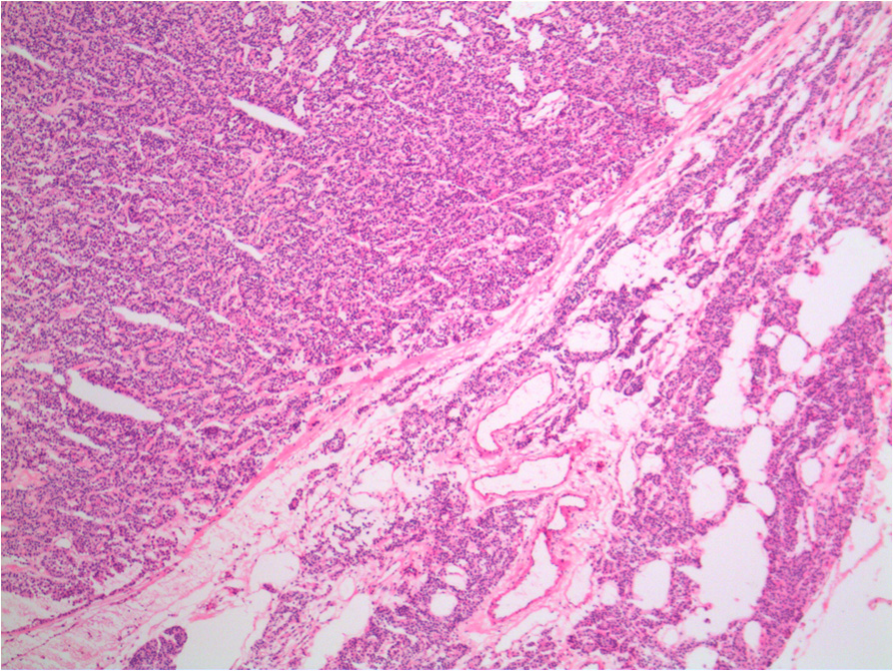
**Interface between parathyroid adenoma and residual normal gland.** The adenoma on the left is hypercellular. The normal gland on the right contains fat.

No statistically significant difference was observed in the incidence of adenoma in their final pathology (*p-*value = 0.168) between hypercellular FS and Adenoma FS groups. Meanwhile, when comparing the above mentioned groups with hyperplasia FS and normal parathyroid tissue FS the groups had a higher incidence of adenoma in their final pathology that was statistically significant (Fisher Exact Test *p*-value <0.001) (Figure 1).

The mean wait time for frozen section was 14.8 minutes (range 9–22 minutes).

#### Intraoperative PTH (IOPTH)

An IOPTH level is considered satisfactory when its value is < 50% of the pre-excision PTH value. In our population, 28 patients did not have a satisfactory IOPTH level. Out of those patients, 10 (35.7%) had a delayed drop in their PTH levels. This drop was noted in the 1-hour postoperative PTH measurement. A second IOPTH level was performed for 11 patients. IOPTH level was useful in 4 patients, all of whom had a final pathology of parathyroid hyperplasia. The management decision intraoperatively for the remaining 15 patients with hyperplasia was based on FS.

Patients with preoperative localizing imaging benefited less from IOPTH compared to patients with non-localizing imaging (1.08% and 8%, respectively) (Pearson Chi-Square *p-*value = 0.017) (Table 2).

**Table 2:** This table demonstrates the reduced usefulness of IOPTH in the group with preoperative 2 concordant imaging localizing the suspected adenoma

**Localization**	**IOPTH not useful**	**IOPTH useful**
Localized	184	2 (1.08% p value 0.017)
Not localized	23	2 (8%)
Total	207	4 (1.9%)

1.08% of the localized group benefited from IOPTH. This was statistically different from the group of patients that did not have 2 concordant preoperative imaging (p value = 0.017).

The mean wait time for IOPTH was 45.7 minutes (range 37 – 68 minutes).

### Operative room added time

IOPTH added an average of 30.9 minutes to each case. This comes to an accumulated added time of 1,138.05 minutes (18 hours and 58 minutes) per year. If IOPTH is to be omitted in patients with preoperatively localized hyperactive gland, this can translate to potentially 1,000 minutes (16 hours and 40 minutes) of saved OR time per year. This figure is estimated based on this study’s preoperative imaging localization rate of 88.15%.

## Discussion

In many centers, it is now accepted that patients with PHPT caused by a solitary adenoma undergo a minimally invasive surgical approach. The advantages of this approach often include a lower complication rate and reduced operative time [10]. IOPTH supports this focused approach by allowing intraoperative confirmation of a successful excision [8,11,12].

In our patient population, the mean age of the patients was 57 years with more prevalence of female gender. Furthermore, the occurrence of parathyroid hyperplasia and double adenomas was 9% and 2.8%, respectively, which are similar to the published value for PHPT. The actual occurrence of hyperplasia, as reported in literature, is 8%-15%, and of double adenomas is around 3% [2,3].

To help in the intraoperative decision in a minimally invasive parathyroidectomy, many surgeons rely on various modalities including preoperative imaging, frozen section, and IOPTH. It is a practice at many centers, including ours, to rely on two concordant imaging to confirm the location of the hyperactive gland preoperatively. Furthermore, frozen section is then introduced to confirm the excision of the hyperactive gland [7]. At our institution, IOPTH is withdrawn at 12 minutes post-excision with using a 50% reduction criteria. Given the half-life of PTH between 4 to 10 minutes, using 12 minutes timing is appropriate [6].

Owing to the advancements in preoperative radiological and nuclear imaging along the improved techniques and experience in frozen sections, researchers and surgeons have started to question the necessity of all these measures to decide on surgery conclusion [13]. As previously mentioned, there are three arms to be addressed preoperative imaging, frozen section, and IOPTH. The scope of this study is focused only on one of the arms i.e., IOPTH, and its need in all cases.

In the present study, 88.15% of the patients had two concordant preoperative imaging modalities localizing a suspected adenoma. This localization had a PPV of 93.5%. It was independent to the modality of imaging studies used (MIBI, US, MRI, CT, or PET Scan). Previous studies reported sensitivity and PPV of a concordant MIBI and US as 95% and 99%, respectively [4].

Similar to others, this study demonstrates that FS is very sensitive to identify an adenoma with sensitivity and PPV exceeding 95% [7]. It is well demonstrated in this research that an FS showing a hypercellular gland or an adenoma carries more than 95% accuracy in being the adenoma.

In our center, IOPTH is being used routinely for 8 years in an attempt to improve the operative success. Nevertheless, this study demonstrates that only 2% of patients with a PHPT had a change in management after the result of the IOPTH. Moreover, patients with a localized adenoma on preoperative imaging had less benefit compared to the group with non-localized (*p-*value = 0.017). These facts raise a question regarding the benefit of IOPTH in the group of patients with a preoperative localized hyperactive gland.

Similar to our results, Bachar *et al.*[4] suggested that patients who had concordant preoperative MIBI and US are likely not to be benefited significantly from IOPTH. Although the value of ultrasound in experienced hands cannot be overemphasized, similar results can be obtained by other concordant imaging modalities such as MRI, if necessary [4].

Furthermore, 35% of the patients who did not demonstrate a reduction in the IOPTH level had a delayed drop as noticed in their 1-hour postoperative PTH measurement (4.7% of 211 parathyroidectomies). These results are similar to previous reports by several authors [4,12]. The latter adds further controversy to the real role of IOPTH and the potential consequences of its use. A false negative IOPTH could influence the surgeon to convert a simple solitary gland parathyroidectomy to 3.5 glands excision [12]. This more aggressive procedure increases risk of recalcitrant hypocalcaemia and of injury to recurrent laryngeal nerve.

Another issue with IOPTH is the added procedure time which varies depending on the protocol implemented and the modality used to analyze the IOPTH. At our center, the mean wait time for IOPTH is 45 minutes with a range that goes as high as 68 minutes. On average, the IOPTH results are obtained 31 minutes after the FS results.

In addition to the consequences of a longer procedure to the patient, IOPTH can also be considered an inappropriate use of the resources. It is especially true in the group of patients with a preoperatively localized hyperactive gland, given that only 1% of patients in this group benefited from it in our study.

On the other hand, IOPTH seems to be of more importance to the group of patients that do not have two concordant preoperative imaging modalities localizing a pathological gland. In this group of patients, 8% would benefit from IOPTH. Although this value is on the lower side, hence caution should be taken in not dismissing the value in this group, as the sample size of the patients who did not have two concordant imaging in this study was small. There is also some importance of IOPTH in cases where preoperative imaging is suggestive of more than one pathological gland to help in differentiating double adenomas from 4-gland hyperplasia.

**Figure 2:**
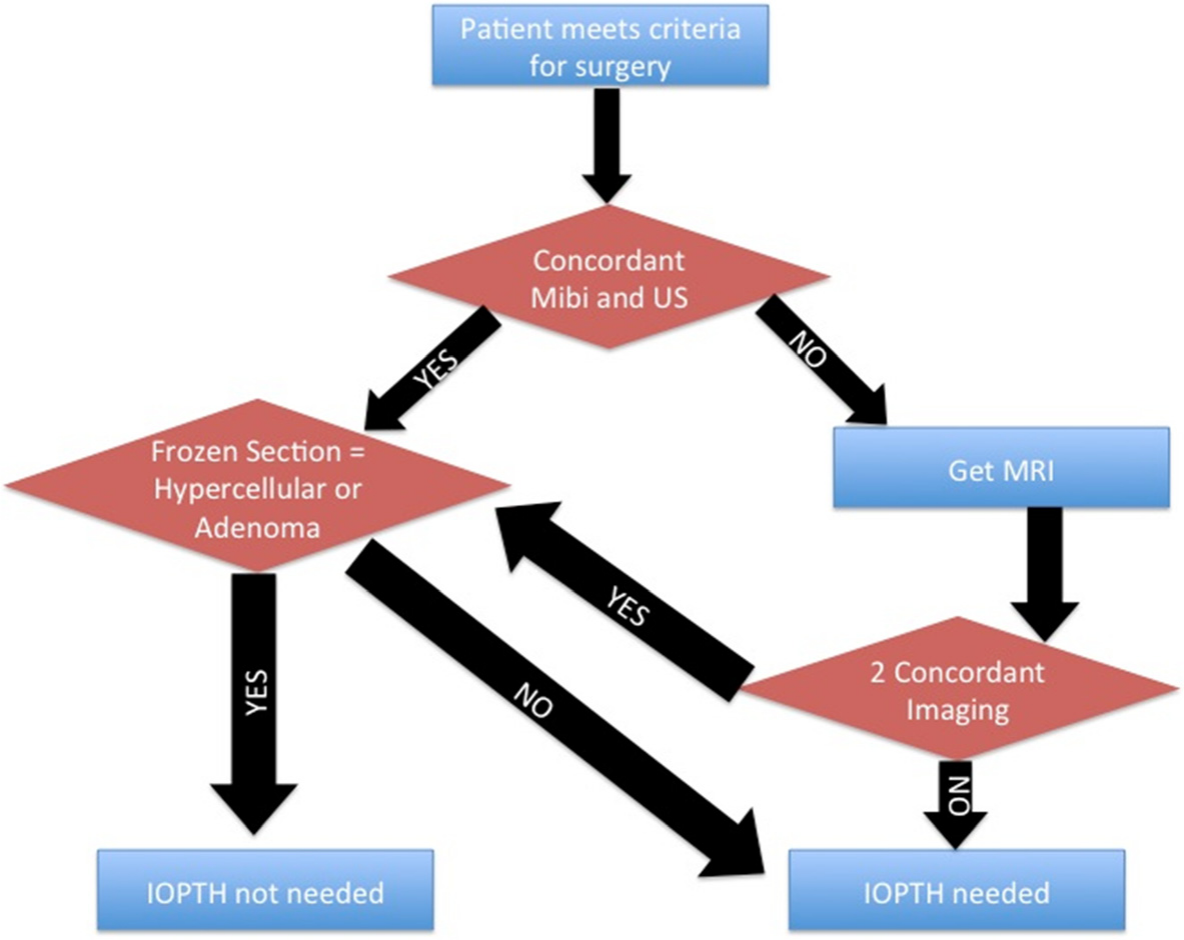
**This figure demonstrates The McGill Parathyroid Protocol (MPP).** This algorithm is applicable for patients with a suspected solitary adenoma.

This study suggests a simple algorithm, The McGill Parathyroid Protocol, to help to approach a patient with PHPT (Figure 2). The use of this algorithm reduces the need for IOPTH by 88%, which translates into decreased need of 4-gland explorations and significantly decreased operative time, while potentially maintains similar success and complication rates.

To our knowledge, this is the first study to identify the group of patients objectively that would benefit from IOPTH with the presence of frozen section and to formulate an algorithm to use IOPTH appropriately.

## Conclusion

IOPTH is a valuable tool in parathyroidectomy, but it has its own set of inherent benefits and challenges. Given the current state of the health care system and limited resources, it may be necessary to limit the use of IOPTH to the patients who would benefit from it more.

Our study demonstrates that patients with localized disease preoperatively benefit from IOPTH if the FS is able to identify a hypercellular gland or adenoma in ~1% of the cases. Whereas 8% of patients, with hyperactive gland that fail to localize, benefit from its use. Implication of The McGill Parathyroid Protocol allows the identification of the appropriate patient and setting to use IOPTH in the presence of frozen section.

## Consent

Written informed consent was obtained from the patients for the publication of this report.

## Competing interests

Authors declare that they have no competing interests.

## Authors’ contributions

Study design: FZ, AMM, MPH, MJB and RJP. Data collection and analyzing: FZ, AC, RV, LR. Drafting the manuscript: FZ, AMM, LR and RJP. Manuscript revision and enhancement: FZ, AMM, RV, MPH and RJP. All authors have read and approved the final manuscript.
